# Planar Microstrip Ring Resonators for Microwave-Based Gas Sensing: Design Aspects and Initial Transducers for Humidity and Ammonia Sensing

**DOI:** 10.3390/s17102422

**Published:** 2017-10-24

**Authors:** Andreas Bogner, Carsten Steiner, Stefanie Walter, Jaroslaw Kita, Gunter Hagen, Ralf Moos

**Affiliations:** Department of Functional Materials, University of Bayreuth, 95447 Bayreuth, Germany; Functional.Materials@uni-bayreuth.de (A.B.); Functional.Materials@uni-bayreuth.de (C.S.); Functional.Materials@uni-bayreuth.de (S.W.); Functional.Materials@uni-bayreuth.de (J.K.); Functional.Materials@uni-bayreuth.de (G.H.)

**Keywords:** microwave cavity perturbation, resonant frequency, radio frequency based gas sensors, zeolites, in operando spectroscopy

## Abstract

A planar microstrip ring resonator structure on alumina was developed using the commercial FEM software COMSOL. Design parameters were evaluated, eventually leading to an optimized design of a miniaturized microwave gas sensor. The sensor was covered with a zeolite film. The device was successfully operated at around 8.5 GHz at room temperature as a humidity sensor. In the next step, an additional planar heater will be included on the reverse side of the resonator structure to allow for testing of gas-sensitive materials under sensor conditions.

## 1. Introduction

Sensors for gas detection include optical gas sensors based on light absorption, metal oxide gas sensors based on a resistive effect (chemiresistors), catalytic gas sensors through adsorption and thermic reaction, gravimetric SAW detectors, gas chromatography, calorimetric devices, biochemical sensors, and many other sensors based on capacitive, amperometric, or potentiometric effects [1]. In typical chemiresistors, gas-sensitive functional films are applied on substrates that are covered with (interdigital) electrodes, and their resistances or their complex impedances are determined [2,3,4]. Mostly, sensors are operated in the range of room temperature to 400 °C. High ohmic electrode–film interfaces and/or very high resistivities of the sensitive materials may exclude promising sensor materials from technical application.

As an alternative approach, devices based on microwave transducers have also been suggested with growing research interest in recent years [5,6,7,8,9].

For sensor purposes, microwaves, especially electromagnetic waves in the range from 1 to 20 GHz, are typically used with planar or hollow waveguides. The sensor effect is the analyte-dependent permittivity of the material. With respect to miniaturization and low-cost applications, planar waveguide-based chemical sensors have attracted recent attention [10]. Several microwave-based sensors have been proposed in the past: moisture detection for the food and chemical industries [11,12,13,14] or glucose detection for biomedical applications [15], to name a few. In the past few years, planar microwave transducers for NH_3_, toluene, CO_2_, or CH_4_ based on the highly sensitive resonant method were suggested [5,6,7,8,9]. Rossignol et al. used coplanar microwave transducers and developed a resonant coplanar structure for detecting NH_3_ and toluene in concentrations up to 500 ppm [7]. They carried out their experiments at room temperature and regenerated the sensitive phthalocyanine layer under argon. Bailly et al. demonstrated a hematite- and a zeolite-based variant of this sensor [8,9]. A concept by Zarifi et al. involves planar resonators and an active feedback loop for a gain that yields up to five times higher quality factors and thus higher resolutions [16]. They also published a zeolite-based version of their high Q resonator for sensing of CH_4_ and CO_2_ concentrations between 1% and 50% [5]. With respect to the sensing aspect, framework materials that adsorb chemical species, like metal-organic frameworks (MOFs) [17] or zeolites [18,19,20], may be preferred for microwave gas sensors range since the adsorption of large amounts of polar gas species goes along with an appropriate change in the complex electrical permittivity. Very recently, Bahoumina et al. proposed a chemical gas sensor with a planar capacitive microwave transducer in the microwave range and a polymer carbon nanomaterial as the sensitive layer [21], and Zarifi et al. demonstrated their active-resonator concept with MOFs as a sensitive material to detect CO_2_ [22]. Another, newer publication of Bailly et al. demonstrated a microstrip spiral resonator covered with titanium dioxide nanoparticles [8]. Recently, further microwave-based sensing concepts with different sensing purposes and planar geometries were published. They include a coupled line section for dielectric sample measurements, interdigital capacitors for liquid mixture concentration measurement, and a disc-shaped tip with a zeolite for thermal mass gas sensing [23,24,25,26]. Even proteins can be detected if the resonator (or the split in a split ring resonator) is functionalized with aptamers [27]. Another biomedical application using an interdigital microstrip capacitor is reported by Rydosz et al. They developed a microwave-based sensor to detect various volatile organic compounds and confirmed that it can be used for exhaled acetone detection [28].

The previous solutions demonstrate the capability of microwave-based sensors to monitor gas concentrations and/or the analyte loading of a sensitive layer. However, research on design and application of microwave transducers for gas detection is in its infancy and needs further investigations to develop integrated sensor devices. In addition, the size of recent devices prevents small applications and dynamic fast measurements with gas sensitive framework layers like zeolites due to their room temperature application. Hence, in this work, a very small 9 GHz resonant microstrip structure with a ring geometry for NH_3_ detection is investigated. As the sensitive material, the ring resonator is coated with an NH_3_ and water adsorbing Fe-zeolite as it is used for selective catalytic NOx reduction reactions in automotive exhausts [29,30]. It is demonstrated that small humidity and ammonia concentrations can be detected. The paper discusses design aspects that were obtained by FEM simulation for planar cavity ring resonators and demonstrates their applicability by fabricating an entire setup and conducting initial measurements at room temperature. We expect that the proposed microwave transducer will open up new possibilities for material characterization and gas-sensing purposes, especially when in the second step a heater layer will be implemented.

## 2. Principle of Planar Microwave Transducers

Due to their interaction with the analyte, the gas sensitive material changes its complex electrical properties of permittivity *ε* = *ε*’ − j*ε*’’ (with j being the imaginary unit) and permeability *µ* = *µ*’ − j*µ*’’. The varying material properties lead to a changing wave propagation. Besides these intrinsic material effects, the wave propagation depends on the material’s geometry and on the design of the used waveguide (extrinsic properties) [10].

**Figure A1:**
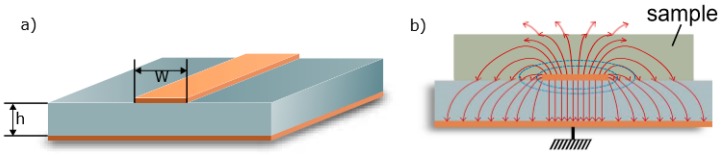
Microstrip waveguide: (**a**) cross section with ground plane and strip conductor; (**b**) electric (red) and magnetic (blue) field distribution with sample.

Generally, electromagnetic waves are guided to a desired transmission mode by restricting their expansion in one or two dimensions. A widely used transmission structure is the planar microstrip line, as depicted in the Appendix A (Figure A1). Microstrip lines consist of a strip conductor and a ground plane separated by a dielectric substrate and thus only support the transversal electromagnetic mode (TEM). TEM modes can be found whenever there are two conductors in only one medium. Hence, to be more precise, the supported wave mode for microstrips is not strictly TEM since the waves propagate not only in the substrates but also in the medium above the conductor line. This causes different phase velocities and, in turn, a longitudinal component of the electric magnetic field. However, since this component is very small it can be often neglected and the supported wave mode is then called quasi-TEM mode. In addition, the two-dimensional structure of microstrips make them well suited for miniaturization and integration with other components and, as a result of the single plane structure, they can be fabricated conventionally by thick or thin film technology, photolithography, photoetching, or laser structuring [31].

Like for microwave-based material characterization methods based on microstrips, non-resonant or resonant methods are applicable, but resonant methods are preferred for sensing applications due to their higher sensitivity and accuracy. Well-known in this respect is the resonant perturbation method, which is based on the resonant frequency change of the scattering parameters by introducing a sample [10]. Examples for such applications in the field of chemical sensors are the characterization of catalyst powder samples in situ during chemical reactions [32,33], or the state monitoring of catalysts directly in exhaust for three-way catalysts (TWC) [34], selective catalytic reduction catalysts (SCR) [35], and diesel particulate filters (DPF) [36]. All are based on the fact that the metallic catalyst or filter housing form an electromagnetic waveguide cavity [37].

Typical planar resonance structures with microstrips are ribbon resonators, disk resonators, or ring resonators [31]. Owing to their geometry, standing waves form. They are trapped between the boundary conditions of the designed microstrips. Microstrip resonators must be coupled with one or two feed lines for excitation. Two types of information can be obtained: the reflection parameters *S*_11_, where only one coupled feed line is required; and the reflection/transmission type. Here, two feed lines are used, allowing evaluation of the reflection parameter *S*_11_ as well as the transmission parameter *S*_21_ [31].

**Figure 1:**
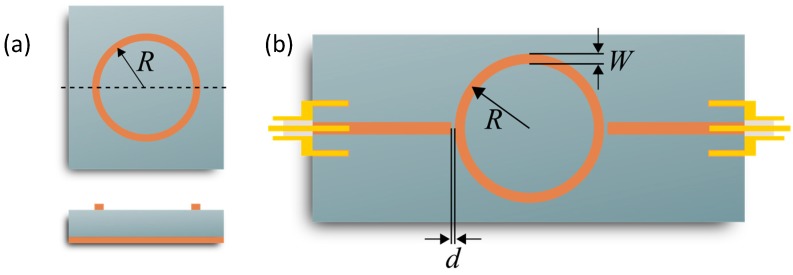
Microstrip ring resonators: (**a**) basic setup and (**b**) transmission-type ring resonator with two capacitively coupled feed lines for excitation.

The standing waves on the structures can be perturbed by a sample. In the case of gas-sensitive layers, the resonance condition is then not only dependent on the permittivity of the substrates, but also from the cover layers, like the here-used zeolites. Then, the effective permittivity *ε*_eff_ describes the combined permittivities of the substrate and gas-sensitive layer. This leads to the resonant frequency *f*_res_:

(1) $$f_{\mathrm{res}}=\frac{c}{L_{\mathrm{ch}}\sqrt{\varepsilon_{\mathrm{eff}}}},$$

where *L*_ch_ denotes the respective characteristic resonance length. It can be obtained by the design equations of Hammerstad and Jensen [31]. The characteristic resonance length depends only on the resonance geometry used. For a ring resonator, *L*_ch_ = 2·π·*R* can be used [31]. For the meaning of the ring resonator radius, see Figure 1. Besides the resonant frequency, the 3dB-bandwidth *BW*_3dB_ is another characteristic parameter of microwave resonators. It leads to the loaded quality factor *Q*, which can denote a measure for the accuracy of the resonance peak [31].

(2) $$Q=\frac{f_{\mathrm{res}}}{BW_{3\mathrm{dB}}}$$

All in all, the transducing principle can then be easily understood. With gases that adsorb in the sensitive layer, the overall complex effective permittivity changes and a resonant frequency and quality factor shift occurs.

## 3. Sensor Design

### 3.1. Microstrip Ring Resonator Design

In this work, a microstrip ring resonator is used. The lack of open ends decreases the radiation losses and thus increases the quality factor compared with open-end resonators. The main design parameters are the coupling gap *d* between the ring resonator and the feed lines; the microstrip width *W* of the ring (both leading to the characteristic impedance); and the mean ring radius *R*, which determines the base resonant frequency (Figure 1). All these parameters influence the electric field distribution at resonance and thus the sensitivity as well as the resolution of the observed resonance peak. To understand their impact, a detailed simulation study was conducted. It is discussed in the following.

### 3.2. Simulation Studies

The simulations were implemented using the RF-module of the FEM software COMSOL Multiphysics. The general structure of the model includes 50 Ω SMA connectors, two 50 Ω feed lines, and the microstrip ring resonator. For the first models, RO4003C (manufactured by Rogers Corp.) specifications were used as a substrate material (substrate thickness *h* = 0.813 mm; permittivity ε’ = 3.38; loss tangent tan *δ* = *ε*’’/*ε*’ = 0.0027).

### 3.3. Influence of the Coupling Gap

To study the influence of the coupling gap, the distance *d* between the ring resonator and the feed lines of a transmission-type microstrip ring resonator was increased. Generally, it is known that a larger coupling gap improves the transferred signal since the electrical field perturbation in the coupling gap is larger for small coupling gaps. Furthermore, it influences the resonant frequency and the resolution of the resonance peak (quality factor). To be more precise, a loose coupling yields a high quality factor and thus a good accuracy (undercoupling), whereas a high coupling level is needed for power transfer (overcoupling) [31]. The required level of coupling is complex and simulation studies of the coupling gap *d* help to identify the influences of coupling gap variations and to estimate needed values. The parameters were varied with the first resonant frequency *f*_res_ = 4 GHz, microstrip width *W* = 1.86 mm, and an impedance of 50 Ω. The smallest coupling gap was set to *d* = 0.025 mm and was increased in 0.025 mm steps to 0.300 mm.

**Figure 2:**
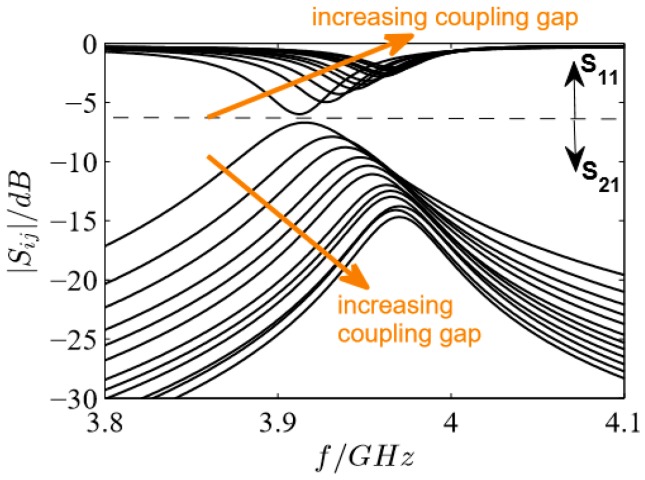
Scattering parameters of the coupling gap simulation study (*d* = 0.025 mm to 0.300 mm in steps of 0.025 mm).

The expected behavior with increasing gap width is confirmed by the simulations in Figure 2. The quality factor is higher (smaller peak width) for loosely coupled resonators and the resonant frequency increases as well due to the perturbation at the coupling gap. Using the reflection parameter *S*_11_, an increased coupling broadens the minimum, i.e., the resonant frequency cannot be as accurately determined. This phenomenon has to be taken into account if the reflection parameter is used as the microwave signal, and the coupling gap *d* has to be selected differently.

### 3.4. Miniaturization

For miniaturized devices, the microstrip resonators have to be operated at higher frequencies. However, then the microstrip losses also increase and the quality factor gets smaller. In addition, a small ratio *W*/*R* is critical due to curving effects that influence the resonator characteristics. Consequences of such small *W*/*R*-ratios may be parasitic resonances as well as higher losses. To account for this effect, another parameter study for different resonant frequencies from 1 GHz up to 10 GHz was conducted. The characteristic impedance (ring microstrip) was kept to 50 Ω, the ring width was set to *W* = 1.86 mm, and the coupling gap to *d* = 0.15 mm. The ring radius *R* was modified between 2.88 mm and 29.2 mm in a way such that resonant frequencies between 1 GHz and 10 GHz occurred. In Table 1, the exact ring radius values and the respective resonance frequencies are listed.

**Figure 3:**
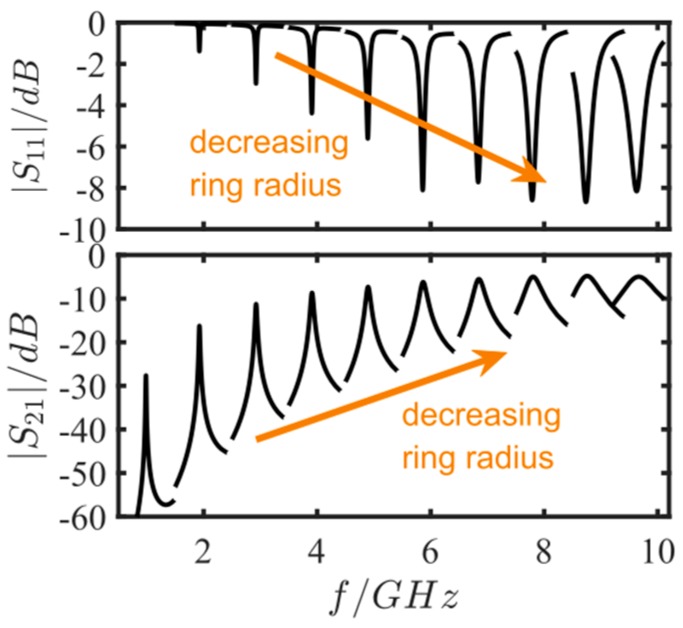
Scattering parameters for the miniaturization simulation study. The ring radius *R* was varied between 2.88 mm and 29.2 mm, so that the resonant frequencies increased from 1 GHz to 10 GHz.

In Figure 3, the results of the miniaturization study are plotted. At higher resonant frequencies, the resonance peaks broaden, i.e., the quality factors get smaller. Furthermore, the transferred signal increases. This could be due to the smaller distances that have to be overcome. Finally, the miniaturization study shows the importance of increasing the quality factor by other parameters to ensure a proper resolution for sensing. Hence, a miniaturization by using high-permittivity substrates should also be considered to improve the sensor signal. Despite the miniaturization improvements, the microstrip substrate has other impacts on the sensor device characteristics. These criteria and the here-used substrate are briefly discussed in the following.

Choosing the substrate plays a key role in the design of microstrip circuits and is widely discussed in literature [38]. Since it is intended to heat up the device in further works, an alumina substrate with a purity of 99.6% and gold as metallization is used. Alumina also ensures a very low loss tangent (*ε*’ = 10.1 @ 1 GHz, tan *δ* = 0.00022 @ 1 MHz, *h* = 0.635 mm; data sheet of CeramTec Rubalit 710). On the other hand, concerning the theory of field distribution in microstrips, the high permittivity negatively affects the sensitivity, since the field concentrates in the substrate with its higher permittivity. However, a higher permittivity allows smaller devices.

### 3.5. Final Microwave Gas-Sensing Device

Generally, a design with only one port is desired for gas detection due to the simplified setup. However, a two-port microwave transducer delivers more information than a one-port reflection-type resonator. Thus, differences between a reflection- and a transmission-type resonator with respect to the sensitivity were simulated. The sensitivity is here defined as a change of the resonant frequency towards a change in the intrinsic material properties of a ring covering layer:

(3) $$S_{\varepsilon }^{f_{\mathrm{res}}}=\frac{\Delta f_{\mathrm{res}}}{\Delta \varepsilon_{\mathrm{eff}}^{\prime }}.$$

For the simulations, the mean radius was set to *R* = 2.07 mm, the covering layer thickness (zeolite) was 0.1 mm, the coupling gaps were set to 0.15 mm, and the Al_2_O_3_ substrate (*ε*’ = 10.1) was chosen to be 0.635 mm. 

**Figure 4:**
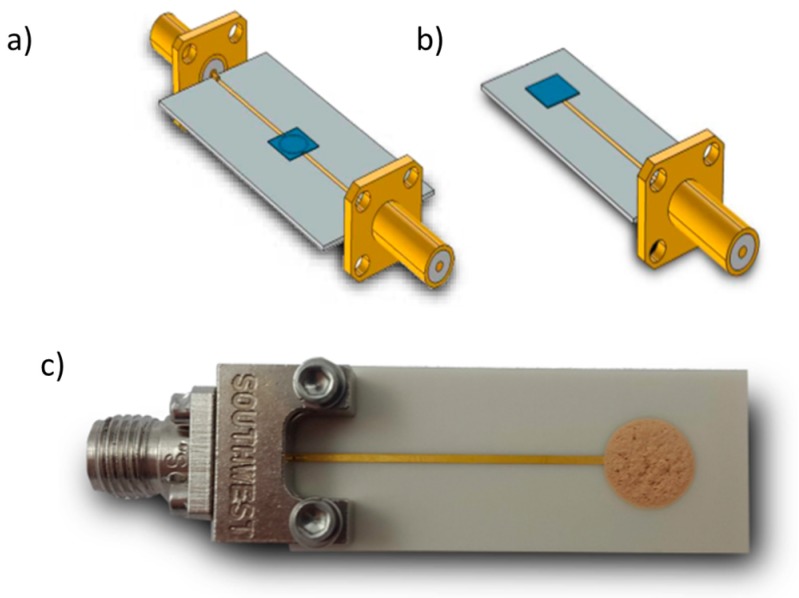
Three-dimensional sensor models with gas-sensitive (zeolite) cover layer (blue) for the sensitivity simulation. (**a**) Transmission-type and (**b**) reflection-type resonator. (**c**) Fabricated microwave ring transducer with Fe-zeolite as sensitive layer and end-mounted SMA connector (specifications: Al_2_O_3_ with 99.6% purity and a thickness of 0.635 mm, *R* = 2.07 mm, *d* = 0.15 mm, *W* = 0.22 mm).

**Figure 5:**
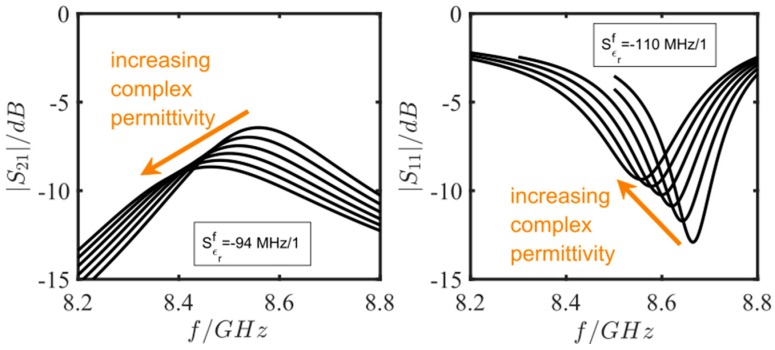
Scattering parameters of the sensitivity simulation with changing complex permittivity from 3 + j0 to 4 + j0.5. Two resonator configurations were simulated: a transmission-type sensor with two excitation lines (2-port measurement, left figure) and a reflection-type sensor with only one excitation line (1-port measurement, right figure). The mentioned sensitivities are calculated from a linear fit of the resonance frequency change.

The setup of both simulated resonator types is depicted in Figure 4a,b. The complex permittivity of the zeolite was increased in the real and the imaginary part to account for the polarization change and a change in intrinsic losses with increasing absorbed ammonia or water [35]. A list of the study steps used is depicted in Table 2. Figure 5 demonstrates that, for the selected data, the sensitivity of the reflection-type device is higher and the resonance peak is less broad than for the transmission-type sensor. Consequently, in the following measurements, the reflection-type resonator was used.

Finally, the coupling gap, microstrip width, and first resonance were chosen based on the performed simulations. To miniaturize the microwave transducer, the resonant frequency of the uncovered ring resonator was set to 9 GHz, which leads to a ring radius of only *R* = 2.07 mm. However, the simulation studies showed that this would lead to a smaller accuracy of the resonance peak. To counter this, the ring strip width was set to *W* = 0.22 mm, which is very small and improves accuracy as well as sensitivity due to the higher field concentration above the substrate for smaller strip widths. The value *d* = 0.15 mm was empirically determined by the preliminary fabricated ring resonators and denotes a compromise between signal intensity and resonance peak accuracy. Finally, the designed microwave ring resonator on alumina substrate was covered with the zeolite. Due to the mechanical instability of the zeolite cover layer, a thickness measurement was difficult. Additionally, the cover layer is not exactly planar, which leads to different thicknesses across the ring resonator. The thickness is assumed to be between 0.5 mm and 1 mm. 

## 4. Experiment and Methods

Al_2_O_3_ substrates were purchased from CeramTec (Rubalit 710, thickness 0.635 mm, *ε*’ = 10.1 @ 1 GHz, tan *δ* = 0.00022 @ 1 MHz) and in-house coated by evaporation with a 400nm gold layer (adhesion layer 4 nm chromium). The microstrip ring, as well as the microstrip feed lines, were structured using a frequency-tripled Nd:YAG-Laser (LPKF Microline 350L, LPKF Laser & Electronics AG, Garbsen, Germany). The laser was also used to drill holes at the ends of the feed lines. They served to fix the end launch (Southwest Microwave, Tempe, AZ, USA) at the ends of the substrate. Both types—the reflection type with one port and the transmission type with two ports—were fabricated this way.

The Fe-zeolite powder (obtained from an automotive catalyst (PSA, further described in [39])) was mixed with a 1:11 compound of ethyl cellulose and terpineol, which was then applied on the microstrip ring and fired at 600 °C (layer thickness between 0.5 mm and 1 mm). After this, the connectors were attached and coaxial cables connected. The entire reflection-type sensor is shown in Figure 4c. This type of zeolite was used because we know its electrical properties in the microwave range from microwave-based measurments in exhaust gas catalysts [39]. 

**Figure 6:**
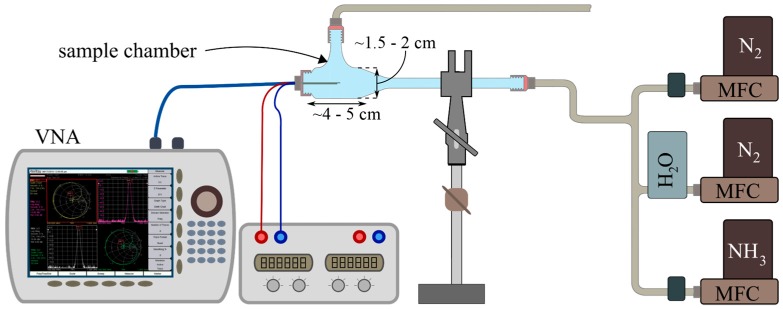
Experimental set-up with glass sample chamber containing the microwave transducer, a vector network analyzer (VNA, Anritsu MS2820B), and a gas supply unit connected to the sample chamber.

The experimental setup included a glass sample chamber containing the microwave transducer, a vector network analyzer (VNA: Anritsu MS2820B), a data acquisition system (Laptop), and a gas supply unit connected to the sample chamber. The latter consisted of three mass flow controllers to control the flows of N_2_, NH_3_, and the water-saturated N_2_. The total gas flow rate was set to 0.5 mL/min. The network analyzer with connected coaxial cables was calibrated using a SOLT calibration kit (Anritsu SMA Calibration Kit 3650). The setup is depicted in Figure 6.

As the sensor response, the reflection parameter *S*_11_ was used. The complex reflection parameter *S*_11_ was measured in a frequency range from 8 GHz to 10 GHz every 10 to 20 seconds. Then, before the resonant frequency and the absolute value of the reflection coefficient (|*S*_11_|) were determined, the reflection spectrum around the operating resonant frequency was filtered using a Lorentz fit to remove noise and parasitic reflections.

Generally, after each adsorption measurement, the zeolite had to be thermally regenerated in an external furnace. Therefore, the coated microwave transducer without the SMA connector was heated up to 120 °C over 2 min and kept at 120 °C for 10 min, then heated up to 600 °C over 25 min and kept at 600 °C for 10 min. Finally, the transducer was cooled down to room temperature over 25 min (≈21 °C). Furthermore, after storing in ambient air, the microwave transducer was purged in N_2_ for several minutes to desorb stored water on the zeolite (see also next section).

## 5. Results and Discussion

### 5.1. Initial Tests: Proof of Concept

**Figure A2:**
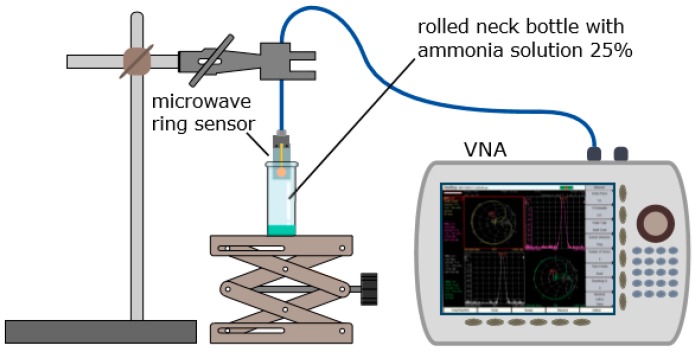
Schematic illustration of the simplified experimental setup for basic verification of adsorption and desorption monitoring with the microwave sensor and a VNA.

Before the measurements in the above-described setup started, initial tests were performed to verify the basic concept and the repeatability of the microwave-based adsorption monitoring of the zeolites at room temperature. For that purpose, the microwave sensor was connected to the VNA and placed in different atmospheric conditions. Apart from ambient air, a rolled-neck glass partly filled with an ammonia-water solution (25 m% NH_3_ in H_2_O) was used (setup in Figure A2). The evaporation of ammonia into the gas phase established an NH_3_ partial pressure in the headspace of the liquid inside the glass, which was used for the first tests. Finally, four different cases were distinguished with this experiment:

1. Sensor outside the glass (i.e., in air) without zeolite layer.
2. Sensor inside the glass (ammonia-in-air ambience) without zeolite layer.
3. Sensor outside the glass (i.e., in air) with zeolite layer.
4. Sensor inside the glass (ammonia-in-air ambience) with zeolite layer.

If the sensor is placed inside the glass (in the headspace of the liquid), both gaseous ammonia and water should adsorb in the zeolite and shift the sensors’ resonant frequency towards lower frequencies since the effective permittivity increases. For reference purposes, the experiment was also repeated without a zeolite cover layer. In addition, the initial test cases were simulated with Comsol Multiphysics. The geometry for the models was set to be the same as the fabricated ring resonator sensor (specifications: substrate thickness of 0.635 mm, mean ring radius *R* = 2.07 mm, coupling gap *d* = 0.15 mm, ring microstrip width *W* = 0.22 mm, zeolite thickness 0.75 mm). The Al_2_O_3_ substrate properties were chosen to be *ε*’ = 9.87 and tan *δ* = 0.0003, which is close to the corresponding datasheet values. Furthermore, there were no data values for the complex permittivity available for the used Fe-zeolite and the ammonia-saturated Fe-zeolite. Thus, the complex permittivities of the zeolites were chosen in a way that they fit the measurement data best (*ε*’_zeolite,air_ = 3.35, tan *δ*_zeolite,air_ = 0.07 and *ε*’_zeolite,NH3_ = 3.97, tan *δ*_zeolite,NH3_ = 0.095). These values can be thought of as a first guess of the actual permittivity of the zeolite and the sensitivity. Roughly they agree with data from [40] for zeolites. Finally, considering the attenuation of transmission lines in the measurement setup, an attenuation of 0.55 dB was added to the Comsol simulation data. 

**Figure 7:**
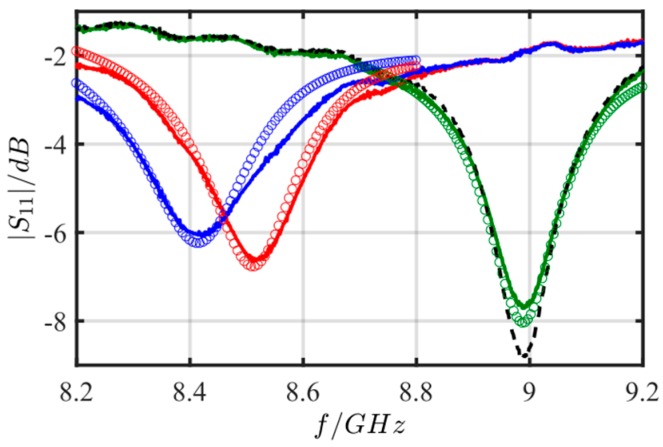
Initial tests: absolute value of the reflection parameter |*S*_11_| of the microwave sensor for different atmospheres: solid line = measurement; circle line = simulation. ① Sensor outside the glass (i.e., in air) without zeolite layer (black dashed); ② Sensor inside the glass (ammonia in air ambience) without zeolite layer (green); ③ Sensor outside the glass (i.e., in air) with zeolite layer (red); ④ Sensor inside the glass (ammonia in air ambience) with zeolite layer (blue).

Figure 7 depicts the measured reflection spectra for the four cases. As expected, all resonance peaks differ from each other. The resonant frequencies without a zeolite layer in air and in ammonia-in-air are identical (① and ②). This was expected, since the gas phase above the sensor hardly contributes to the resonant frequency due to their low permittivity. As soon as the microstrip ring is covered with a zeolite layer, the permittivity of the zeolite forces the fundamental resonant frequency to shift to lower frequencies (③). Furthermore, by exposing the zeolite-covered sensor to the ammonia-in-air ambience, a further decrease to lower frequencies can be observed, which can be attributed to the permittivity increase when ammonia and/or water adsorbs in the zeolite [41,42,43]. From in situ experiments with zeolites applying the cavity perturbation method, the general relation between ammonia adsorption and the electromagnetic material properties have already been studied [32,35].

**Figure 8:**
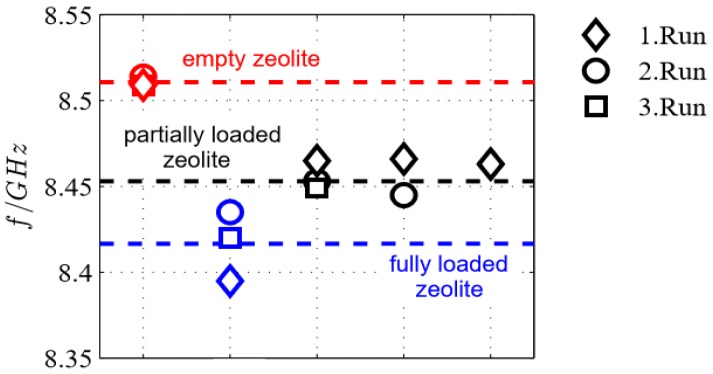
Initial tests: resonant frequencies of the microwave sensor for the three experimental runs with different loading conditions of the zeolite: empty, partly loaded, und fully loaded (for further details see text).

The zeolites were regenerated and the experiment was repeated another two times. For each run, the resonant frequency for the unloaded case (after regeneration in air), fully ammonia-loaded case (ammonia-in-air ambience), and now also the partially loaded case (in air after exposing to ammonia) were recorded and compared. For the latter, the fully loaded zeolites were again exposed to air. Thereby, ammonia (defined as weakly bonded ammonia [44]) desorbs until a new equilibrium is established and only strongly bonded ammonia remains in the zeolite. The respective resonant frequencies are illustrated in Figure 8. It should be noted that the experiments were carried out at different days and thus at different room temperatures as well as humidity. Nevertheless, in Figure 8, the resonant frequencies at the time *t*_0_ for the empty zeolite coincide. Additionally, for all experimental runs the same trends can be seen in the frequency shift. The quantitative differences may be explained by the varying experimental differences in humidity and room temperature. Furthermore, the unknown ammonia and water concentrations make an explanation difficult. Therefore, for the subsequent tests, the above-described sample chamber with the gas supply unit and defined concentrations was used.

### 5.2. Tests under Defined Gas Exposure

**Figure 9:**
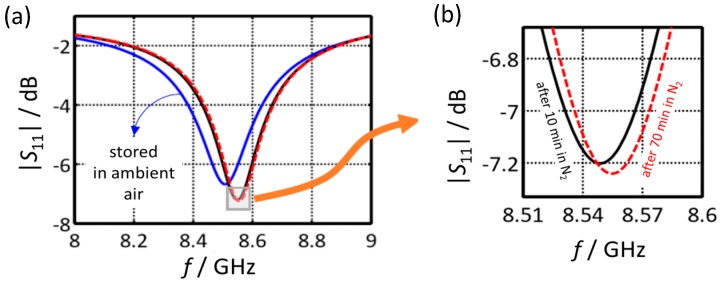
Absolute value of the reflection parameter |*S*_11_| of the microwave sensor when stored in ambient air and when purged with nitrogen: (**a**) overall response from 8 to 9 GHz (**b**) near resonance response.

Since humidity in air should affect the resonant frequency, magnitude and timely response behavior were observed during water desorption. A sensor that was stored in air was installed in the test rig as described in Figure 6 and was then purged with pure N_2_. The respective reflection spectra around resonant frequency are shown in Figure 9. The blue curve denotes a spectrum after storage in air. The black and the red curve were recorded after purging with N_2_. One can clearly see that both *f*_res_ and |*S*_11_| are affected due to the incorporation of water into the zeolite during storage in ambient air. Since water increases the permittivity of the zeolites, the resonant frequency increases after the desorption of water. The results also show that after 10 min most of the water has already desorbed, even at room temperature (Figure 9b). On the other hand, it is clear that before each measurement, the sensor has to be purged for several minutes to obtain reproducible results. 

**Figure 10:**
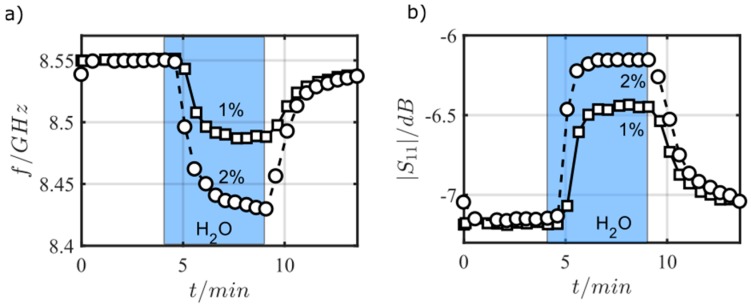
Resonant frequency (**a**) and reflection parameter at resonant frequency (**b**) when exposed to pure N_2_ and to 1 vol % water or to 2 vol % water in N_2_.

Then, the influence of defined water content was investigated. Figure 10 is a time-dependent plot of *f*_res_ and |*S*_11_|@*f*_res_ for an admixture of 1 vol % and 2 vol % of water into nitrogen. One can clearly see that the device responds to water. This excludes O_2_ or CO_2_ (both from air) from being responsible for the sensor effect in Figure 9. Again, adsorbed water can be desorbed in water-free nitrogen even at room temperature, but the process requires a longer time. Here, higher temperatures would be favorable.

**Figure 11:**
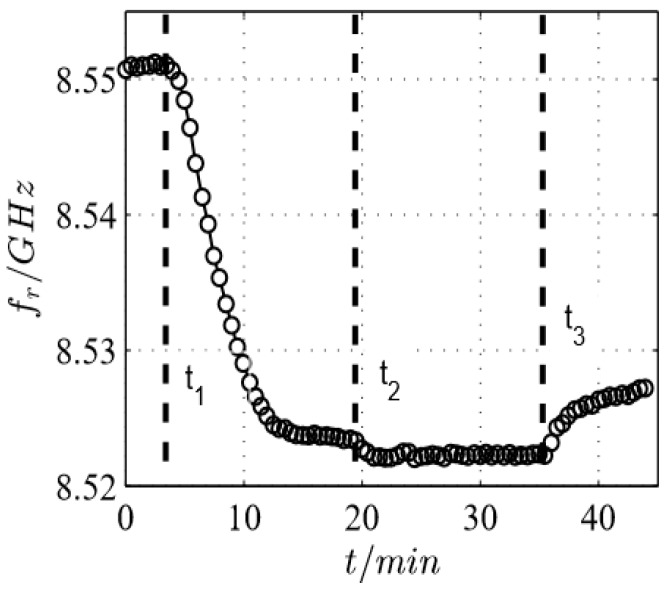
Time dependency of the resonant frequency of the microwave sensor according to Figure 4c when 500 ppm (*t*_1_) or 1000 ppm NH_3_ (*t*_2_) are added to N_2_. At *t*_3_, NH_3_ was turned off.

In further experiments, defined NH_3_ concentrations were admixed. The sensor was exposed to a stepwise-changing NH_3_ concentration from 0 ppm to 500 ppm (*t*_1_) and from 500 ppm to 1000 ppm NH_3_ (*t*_2_) without regenerating the zeolite in between. Again, the obtained results in Figure 11 show different resonant frequency shifts during the loading with 500 ppm and 1000 ppm NH_3_. In the first measurement step between *t*_1_ and *t*_2_, when loading with 500 ppm NH_3_, it can be clearly seen that the sensor signal is accumulating until saturation with ammonia is reached. Furthermore, increasing the ammonia concentration to 1000 ppm also leads to a distinguishable but smaller resonant frequency shift. The differences in the sensor signal between the 500 ppm saturation and the 1000 ppm saturation is due to the higher partial pressure of NH_3_ that causes a shift towards the adsorbed phase in the adsorption isotherm. The feed with 1000 ppm ammonia is finally stopped and the gas sensor test chamber is purged with pure N_2_ (*t*_3_). Consequently, the resonant frequency shift decreases, but not to the initial value as probably expected from water desorption. It is assumed that only so-called weakly bonded NH_3_ can desorb at room temperature. Strongly bonded ammonia remains on the zeolite even in a pure nitrogen atmosphere at this low temperature. These results agree well with data obtained for >200 °C for an H-ZSM5 zeolite obtained in an electrical in situ cavity resonator [40], if one extrapolates their data to room temperature. However, with respect to gas-sensing applications, it is clear that one needs a higher temperature at which the desorption is fast enough to guarantee a reversible sensor behavior. Therefore, it is mandatory to establish a heater layer on the sensor substrate. This will be the next step during the sensor development. 

## 6. Conclusions and Outlook

This work investigated the design parameters of a microstrip ring resonator with an Fe-zeolite as a gas-sensitive layer. Based on FEM simulation results and commonly used microstrip design equations, a planar microstrip ring resonator structure on alumina was developed and covered with a Fe-zeolite film. Finally, the device was successfully operated at around 8.5 GHz as a humidity sensor and for ammonia-loading detection at room temperature. The amount of stored water and ammonia in the zeolite is mirrored by both the absolute value of the reflection coefficient at resonance and by the resonant frequency itself. Due to the better resolution, we conclude that the resonant frequency is a suitable measure for ammonia and water detection with this planar device. Furthermore, the concentration-dependent frequency shifts during loading illustrate the accumulating characteristics of the sensor signal and make it conceivable to measure small amounts of gas concentrations by using the device operating in the so-called dosimeter-mode [45]. 

In future work, an integrated heating element will be added to the device. Then it may work as a fast and reversible gas sensor. In addition, it may help to determine adsorption isotherms. In combination with in situ DRIFT spectroscopy, the effect of distinct adsorbed species on the dielectric behavior of the gas sensitive material can also be studied in a wide temperature regime.

## Figures and Tables

**Table 1:** Ring radius *R* to obtain resonant frequencies *f*_res_ between 1 and 10 GHz. The ring width *W* was set to 1.86 mm.

$f_{\mathrm{res}}$/GHz	1	2	3	4	5	6	7	8	9	10
*R*/mm	29.2	14.6	9.72	7.28	5.81	4.84	4.14	3.62	3.21	2.88

**Table 2:** Data for the sensitivity simulation. The complex permittivities were roughly estimated from [32].

Step	1	2	3	4	5	6
*ε*’	3 + j0	3.2 + j0.1	3.4 + j0.2	3.6 + j0.3	3.8 + j0.4	4 + j0.5

## Appendix A

Field distribution in a microstrip waveguide.

Simplified setup for the initial measurements.
